# Full recovery of ultrafast waveforms lost under noise

**DOI:** 10.1038/s41467-021-22716-w

**Published:** 2021-04-23

**Authors:** Benjamin Crockett, Luis Romero Cortés, Saikrishna Reddy Konatham, José Azaña

**Affiliations:** Institut National de la Recherche Scientifique – Énergie Matériaux Télécommunications (INRS-EMT), Montréal, QC Canada

**Keywords:** Imaging and sensing, Optical metrology

## Abstract

The ability to detect ultrafast waveforms arising from randomly occurring events is essential to such diverse fields as bioimaging, spectroscopy, radio-astronomy, sensing and telecommunications. However, noise remains a significant challenge to recover the information carried by such waveforms, which are often too weak for detection. The key issue is that most of the undesired noise is contained within the broad frequency band of the ultrafast waveform, such that it cannot be alleviated through conventional methods. In spite of intensive research efforts, no technique can retrieve the complete description of a noise-dominated ultrafast waveform of unknown parameters. Here, we propose a signal denoising concept involving passive enhancement of the coherent content of the signal frequency spectrum, which enables the full recovery of arbitrary ultrafast waveforms buried under noise, in a real-time and single-shot fashion. We experimentally demonstrate the retrieval of picosecond-resolution waveforms that are over an order of magnitude weaker than the in-band noise. By granting access to previously undetectable information, this concept shows promise for advancing various fields dealing with weak or noise-dominated broadband waveforms.

## Abstract (web summary)

Extracting weak, ultrafast single-shot waveforms from a noisy background has critical uses in many applications. Here, the authors present a method to enhance signals and recover arbitrary waveforms from very noisy backgrounds by manipulating the physical waveform spectra in real time.

## Introduction

Ultrafast or broadband temporal waves, from the microwave to the optical domains, are important to numerous applications. For example, in molecular imaging and biology, ultrafast optical pulses are used to probe deep inside brain tissues^1,2^ and to investigate the dynamics of molecular processes^3^. In information and communication technologies, broadband waves are necessary for transferring massive amounts of data^4^ and for sensing and ranging in pulsed radars or lidars^5^. Broadband waveforms also occur naturally, such as the short radio bursts produced by distant pulsars^6,7^ or the sudden and unpredictable radiation emitted from a decaying molecule^8^. All of these applications fundamentally depend on the capability to detect the relevant ultrafast waves and gain access to the information they may carry. During the past few decades, notable progress has been made on ultrafast signal detection, enabling the measurement, evaluation, and manipulation of waveforms at unprecedented time scales, down to the femtosecond regime^9^. However, despite these advances, stochastic noise is unavoidably introduced onto the signals of interest during their generation, transmission, or detection processes^10^, often preventing retrieval of critical information contained within these waveforms. The problem is especially significant across the above-mentioned applications because the relevant signals are purposely attenuated or naturally weak. For instance, in bioimaging^1^ and lidar systems^5^, optical signals consisting of only a few photons are used, making the related detection processes very sensitive to noise. This represents a hurdle for further progress to fundamental sciences and technologies that operate at the limits of ultra-weak or noise-dominated signal detection.

Typical noise mitigation techniques for weak-signal detection include one or combinations of the following: (1) active amplification, (2) signal averaging, (3) bandpass filtering, and/or (4) computational post-processing. However, none of these methods can recover a non-repetitive broadband waveform lost under noise. Active amplification, by which external energy is introduced into the signal, can be used for boosting and detecting weak, but not noise-dominated, waveforms. The reason is that the active gain process will act on both the waveform of interest and the associated noise, while further injecting its own noise, thus failing to enhance detectability^11^. In some cases (e.g., for repetitive waveforms), signal averaging methods may improve the result of detection; however, in general, the signal to be denoised is an arbitrary non-repetitive waveform, which may occur in an isolated fashion and at any given unknown time, rendering averaging approaches inadequate. Alternatively, bandpass filtering can mitigate noise by attenuating all components outside of the frequency band occupied by the signal of interest. However, this option assumes that the central frequency and spectral extension (bandwidth) of the signal are known a priori, which may not be the case for an arbitrary, random waveform. More importantly, this solution is largely ineffective in the case of broadband signals, because an important portion (if not the majority) of the undesired noise occurs within the inherently wide frequency band of the ultrafast waveform itself. This key in-band noise contribution is especially difficult to deal with, and it has been the subject of intensive research in diverse fields^12–18^. Specifically, digital post-processing methods have proven to be efficient for some narrow-band applications, such as electroencephalogram measurements of brain activity^15^, speech recognition^16^, mechanical monitoring^17^, and image processing^18^. However, these methods are computationally demanding and thus ill-suited for the detection of randomly occurring ultrafast waveforms requiring continuous monitoring. Furthermore, when the signals have frequency bandwidths just above a few tens of GHz, corresponding to temporal variations below the sub-nanosecond range, the digitalization step represents a serious obstacle for practical implementation of such post-processing approaches^19^.

Alternative analog-processing strategies have been suggested for noise mitigation of ultrafast signals^12–14^. However, to our knowledge, none of the methods reported to date can actually recover an unknown waveform of interest lost under in-band noise. In a recent advancement, a sophisticated nonlinear-optics-based scheme was demonstrated to increase the probability of identifying the presence of a pulsed waveform buried under noise^14^ but without providing any further information about the detected event (temporal or spectral shape, duration, central frequency, bandwidth, etc.). This clearly shows the difficulty of the problem at hand. Thus, the recovery of arbitrary (e.g., randomly occurring) ultrafast signals lost under noise remains a crucial and challenging problem to be solved.

Here we present a concept for the full complex-field recovery of subnoise, broadband, non-repetitive signals. Our technique denoises the waveform of interest, even when the signal is totally buried under noise, regardless of its time of arrival, central frequency, bandwidth, or shape. No a priori knowledge of any of these parameters is needed, as desired for most practical applications. Moreover, the waveform denoising process is implemented directly in the analog physical wave domain, thus avoiding the need for any digital post-processing. Referred to as the spectral Talbot array illuminator (S-TAI), the proposed scheme redistributes the frequency spectrum of the incoming signal into discrete peaks whose envelope follows an amplified copy of the waveform of interest over the stochastic (incoherent) noise background. This is achieved through a suitable combination of linear wave phase transformations, namely, dispersive propagation and temporal phase modulation. Using standard telecommunication components, we experimentally show the unprecedented capabilities offered by the S-TAI concept through demonstrating real-time and single-shot recovery of broadband (picosecond resolution) optical waveforms that are entirely buried under in-band noise. We successfully recover waveforms extending over a full-width frequency bandwidth up to ~400 GHz, corresponding to temporal variations as fast as ~2 ps. Furthermore, we also show that the S-TAI method preserves the spectral phase information, such that the temporal waveform representation can be also retrieved faithfully and with the needed high resolution. The principle is based on a combination of linear wave transformations, which are available on virtually all wave systems^20^. This includes both classical and quantum systems over most regions of the electromagnetic spectrum, as well as other physical supports (e.g., acoustics or matter waves), allowing for a wide spread of possible applications.

## Results

### Theory and design principle

**Fig. 1:**
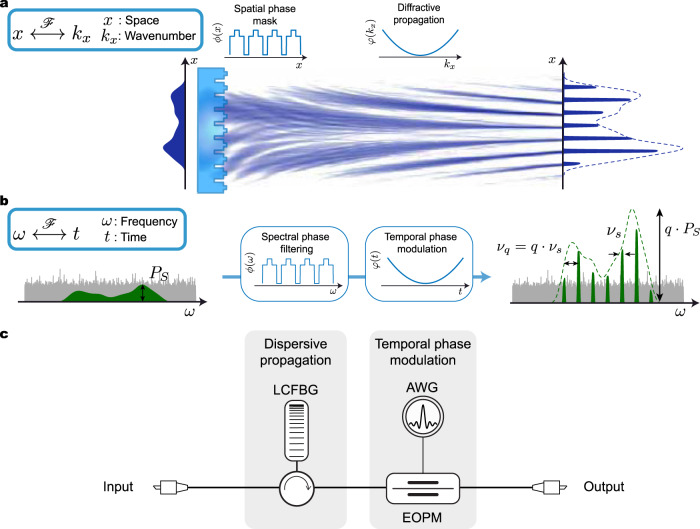
S-TAI denoising concept. **a** Originally observed in the spatial domain, a Talbot Array Illuminator (TAI) focuses a uniform beam into an array of bright spots^21^. A TAI consists of a discrete phase mask *ϕ(x)* applied along the transversal spatial direction *x*, followed by free-space diffraction, which imposes a continuous quadratic phase *φ(k*_*x*_*)* along the angular frequency variable *k*_*x*_, the Fourier-conjugate of *x*^26^. The involved Fourier transformation is indicated by the symbol ℱ. We show here that the well-known TAI mechanism can be applied on a non-uniform spatial wavefront. **b** Through a mathematical analogy between the space and temporal frequency domains, a similar process is implemented along the frequency spectrum representation of a temporal waveform (i.e., S-TAI). In this case, the observation domain is along the radial frequency variable *ω*, and its Fourier-dual domain is along the time variable, *t*. Thus, the S-TAI can be constructed by a suitable discrete spectral phase filter *ϕ(ω)*, followed by a continuous quadratic temporal phase modulation *φ(t)*, as detailed in the main text. This creates a sampled version of the input spectrum, with peaks of width *ν*_*s*_ separated by *ν*_*q*_ = *qν*_*s*_, outlining a copy of the waveform of interest amplified by a factor *q*. On the other hand, the incoherent noise content (here illustrated as a gray background) is left untouched, thus enabling the recovery of a waveform initially buried under noise. **c** Experimental realization of the concept for optical waveforms, with the acronyms defined in the text. For convenience, the S-TAI mechanism can be implemented through a continuous quadratic spectral phase transformation (dispersive phase filtering), followed by a periodic discrete temporal phase modulation process (see “Methods”).

As illustrated in Fig. 1, the proposed noise mitigation concept exploits a frequency-domain analog of a spatial TAI^21^. The TAI was first proposed in the spatial domain as an efficient way to transform a uniform beam of light into a collection of localized bright spots. Rather than using an energy-inefficient amplitude mask to form an array of spots by simply discarding the excess light, the TAI relies on a spatial phase mask that imprints a specific phase shift at different locations along the incoming wavefront. When followed by spatial diffraction through a suitable propagation distance, the light is focused into bright spots in a lossless manner, see Fig. 1a. The concept of a TAI is related to that of an array of lenses where a uniform light beam is concentrated into a set of isolated spots. However, these methods typically offer a limited compression factor due to the maximum focusing power achievable by a single lens^22,23^. On the other hand, a TAI allows for higher compression factors by discretizing the otherwise continuous quadratic phase of the lens elements to specific values derived from number theory^24,25^, so that the phase modulation applied on the uniform wavefront can be restricted to a maximum excursion of 2*π*. Using the well-known space–time duality paradigm^26^, TAIs have also been described in the temporal domain to transform a constant amplitude waveform (e.g., a continuous wave) into a series of short pulses^23^, with applications ranging from optical pulse generation to temporal invisibility cloaking^27^. In this present work, we exploit the fact that a TAI can be implemented on a waveform with an arbitrary shape (i.e., not restricted to application on a uniform wave), with the key finding that the TAI process preserves both the amplitude and phase profile of the incoming waveform through the envelope of the resulting output peaks. By exploiting this feature, we have recently shown that the time-domain implementation of a TAI offers unique abilities for out-of-band noise mitigation of arbitrary temporal waveforms^28^, although it does not allow for in-band noise mitigation. On the other hand, here we show that by implementing the TAI along the frequency-domain representation of the signal (i.e., S-TAI), efficient in-band noise mitigation of broadband arbitrary waveforms can be achieved. In this case, the S-TAI can be interpreted as a lossless sampling process of the signal’s frequency-domain representation, in which the energy spectrum of the input waveform is redistributed into a periodic set of narrow spectral peaks. Since the frequency components of the random noise present along the signal are uncorrelated with respect to each other, this incoherent background noise will be left essentially untouched by the S-TAI phase operations. In sharp contrast, the correlated portions of the signal, i.e., those corresponding to the target phase-coherent waveform, will coherently add into narrow frequency peaks, amplifying the spectral envelope of the coherent waveform of interest, with proportionally lower relative in-band noise contribution.

A spatial TAI is typically implemented using a discrete spatial phase modulation mask along the transverse direction *x*, followed by free-space diffraction. The latter operation imposes a continuous quadratic phase variation on the angular wavenumbers, *k*_*x*_, the variable of the angular spectrum domain, which corresponds to the Fourier dual representation of the transverse spatial domain. Toward realization of the proposed S-TAI concept, an analogy is considered between space (*x*) and radial frequency (*ω*), such that the space and angular spectrum phase operations in the original TAI are mapped into the spectral and temporal representations of a time-varying signal, respectively. Hence, as illustrated in Fig. 1b, in exact analogy to the spatial case in Fig. 1a, the S-TAI can then be implemented by imposing a discretized spectral phase mask on the input signal (i.e., along the frequency variable *ω*) followed by a continuous quadratic temporal phase modulation (i.e., along the time variable *t*). We note that the order of the domains of operation is important here, i.e., first a phase manipulation in the spectral domain is needed, followed by the necessary phase manipulation in the temporal domain. This can be understood by considering that a phase manipulation in a given domain does not alter the intensity profile of the wave distribution in the affected domain, whereas the wave distribution in the Fourier-related domain is altered in a complex manner, typically in regard to both its intensity and phase profiles. Recently, it has been also shown that the TAI fundamentally relies on specific numerical properties of quadratic sequences^24^, such that the basic implementation of a TAI relies on purely discrete quadratic phase manipulations in the two involved domains (see “Methods”). In practice, the implementation of a TAI is, however, fortunately versatile, such that one of the two phase manipulations can be made continuous rather than discrete while maintaining the energy redistributing properties of the TAI process, as long as the manipulation in the other domain is done in a discrete fashion. Thus, since in practice discrete spectral phase filters are challenging to design and offer poor flexibility, the required quadratic spectral phase filtering operation can be instead implemented in a continuous fashion, e.g., through the use of widely available group velocity dispersion (GVD). This process involves wave propagation through a transparent medium in which different frequency components propagate with a group delay that varies linearly as a function of radial frequency^20^. Therefore, for experimental convenience, an S-TAI may be implemented by propagation of the signal of interest through a suitable GVD medium, implementing the needed continuous quadratic spectral phase filtering, followed by discrete multilevel temporal phase modulation (see Fig. 1c and “Methods”).

For implementation of an S-TAI aimed at the generation of output spectral peaks of width *ν*_*s*_ separated by *ν*_*q*_ = *qν*_*s*_, where *q* is the target amplification factor, the waveform is first made to accumulate a total linear GVD ${\ddot{\phi }}_{\,}$ (defined as the slope of the wave’s group delay as a function of radial frequency) satisfying

1 $$\begin{array}{c}\ddot{\phi }=\frac{1}{q}\frac{1}{2\pi {\nu }_{s}^{2}}.\end{array}$$

The corresponding spectral phase filtering process causes the waveform to be temporally broadened and exhibit a quadratic temporal phase variation. The S-TAI is formed by subsequent temporal phase modulation of the dispersed signal according to a periodic discrete pattern that consists of *q* phase steps^24,25,29^, each of duration *t*_*s*_ = *1*/*ν*_*q*_ (see Supplementary Fig. 1), where the *n*th phase step is given by

2 $$\begin{array}{c}{\varphi }_{n}=-\pi \frac{q-1}{q}{n}^{2}.\end{array}$$

The output from this S-TAI design will thus correspond to a spectrally sampled version of the input waveform, in which the resulting spectral peaks have a width *ν*_*s*_ and a separation *ν*_*q*_ as well as an intensity that is locally increased by a factor *q* with respect to the input waveform.

As with any sampling process, to fully recover a complex spectrum shape where the narrowest spectral variations (in amplitude and phase) have a resolution *ν*_*N*_, corresponding to a waveform with a temporal duration shorter than *t*_*N*_ ≈ *1*/*ν*_*N*_, one would need to design an S-TAI where the frequency peak separation satisfies *ν*_*q*_ < *ν*_*N*_, as per the Nyquist criterion^20^. The S-TAI is therefore limited in regard to the maximum duration of the waveform it can recover. However, there is no fundamental limit on the finest temporal resolution that an S-TAI can achieve. In practice, this is simply constrained by the operation frequency bandwidth of the employed components (e.g., dispersive medium or temporal modulation process).

### Experimental demonstrations

The S-TAI concept is here experimentally demonstrated on ultrafast optical waveforms. For this purpose, as illustrated in Fig. 1c, the S-TAI was implemented using a linearly chirped fiber Bragg grating (LCFBG), acting as the dispersive medium, followed by an electro-optic phase modulator (EOPM) driven by an electronic arbitrary waveform generator (AWG) to perform the required temporal phase modulation on the dispersed optical signal (see “Methods” and Supplementary Figs. 2 and 3).

**Fig. 2:**
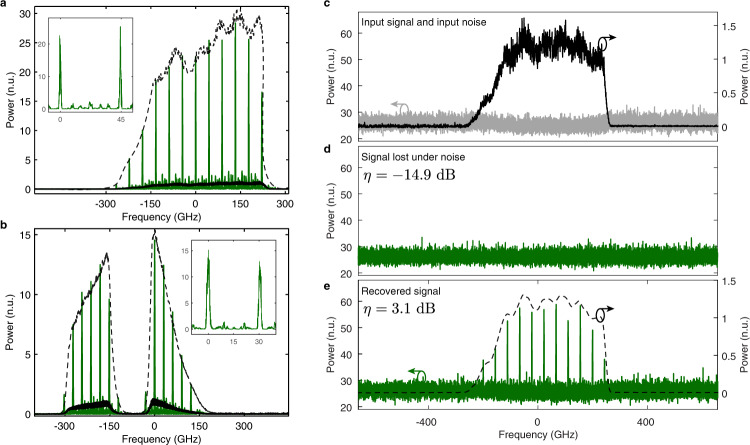
S-TAI amplification and recovery of spectral waveforms. **a**, **b** The input waveform (black) is simultaneously amplified and sampled using the S-TAI (output shown in green). The input waveform scaled by the measured amplification factor is shown for comparison (dashed black trace). **a** The system was first configured for a high amplification factor of *q* = 32, with a peak separation *ν*_*q*_ = 44.8 GHz and a peak width *ν*_s_ = 1.4 GHz (see figure inset). The output waveform resulted in a measured amplification of 29.1 with *ν*_*q*_ = 44.4 GHz, and *ν*_*s*_ = 1.6 GHz. **b** To recover a more complicated waveform (i.e., with a longer duration), the temporal phase modulation signal was electronically reconfigured for *q* = 15, allowing for a higher spectral resolution with a peak separation *ν*_*q*_ = 30.1 GHz and a peak width *ν*_*s*_ = 2.0 GHz. The output waveform resulted in a measured amplification of 14.43 with *ν*_*q*_ = 30.3 GHz, and *ν*_*s*_ = 2.2 GHz. **c** A weak optical waveform (black, right axis) is combined with strong stochastic (ASE) noise (gray, left axis). Notice the significant difference in the vertical scales. **d** The waveform is completely buried under noise, becoming undetectable. **e** Using the same parameters as those in Fig. 2a, the waveform of interest is successfully recovered from noise (green, left axis), as indicated by *η*, a measure of the visibility of the waveform against the noise, see Eq. (3). The input (dashed black trace, right axis) is shown for comparison, confirming that the envelope of the S-TAI peaks outlines an amplified, high-fidelity copy of the input signal. All shown optical spectra are represented as a function of the relative frequency with respect to a center optical frequency of 193.470 THz (wavelength of 1549.56 nm) on a linear scale normalized to the average power of the input coherent waveform measured at the locations of the output peaks (see “Methods”).

To demonstrate the potential for high amplification of broadband arbitrary waveforms, we first designed the S-TAI for amplification by a factor *q* = 32 of a waveform with a full width at half maximum (FWHM) frequency bandwidth of 400 GHz, depicted in Fig. 2a. Using the same components, we then reconfigured our system for a shorter peak separation by simply programming a different temporal phase modulation pattern in the AWG (results shown in Fig. 2b). The narrower peak separation enables a higher spectral resolution, thus allowing to process more complicated waveforms (i.e., longer temporal duration). This exemplifies the versatility offered by the S-TAI to customize the processing system specifications. The capability for recovering signals of arbitrary shape, regardless of central frequency or bandwidth, is further demonstrated through the results shown in Supplementary Figs. 4–6.

The noise mitigation capabilities of the S-TAI are demonstrated through the results in Fig. 2c–e. A large amount of amplified spontaneous emission (ASE) noise is injected into an input coherent optical waveform using an Erbium-doped fiber amplifier (EDFA). We quantify the detectability of the waveform by defining the visibility *η* as the mean of the ratios of the power of the waveform of interest *P*_*S*_ to that of the noise injected by the EDFA *P*_*N*_, measured at the frequency location of each output S-TAI peak relative to the noise floor of the input waveform (see “Methods”), expressed in decibels:

3 $$\begin{array}{c}\eta =10\,{{{\log }}}_{10}\left(\frac{{P}_{S}}{{P}_{N}}\right).\end{array}$$

As shown in Fig. 2c–e, the inserted noise was on average over 20 times stronger than the waveform of interest, resulting in a low visibility of −14.9 dB (see Fig. 2d), such that the signal was entirely lost under the noise background. Using the same parameters as in Fig. 2a, the S-TAI allowed for an increase of the visibility by 18 dB, almost two orders of magnitude. The output spectral waveform could then be recovered from the noise background and adequately measured, accurately depicting a sampled version of the original waveform.

To prove the single-shot particularity of the S-TAI, we employed the well-established dispersion-induced real-time optical Fourier transform (RT-OFT) method^30,31^ (see Supplementary Fig. 3). This technique enables a mapping of the frequency information of an input waveform along the time domain, making the spectrum of a single incoming waveform directly accessible on a real-time oscilloscope (RTO). In our experiments, an amount of dispersion is utilized such that the output spectrum from the S-TAI is mapped along the time domain at a rate of 9.72 GHz/ns (see “Methods”).

**Fig. 3:**
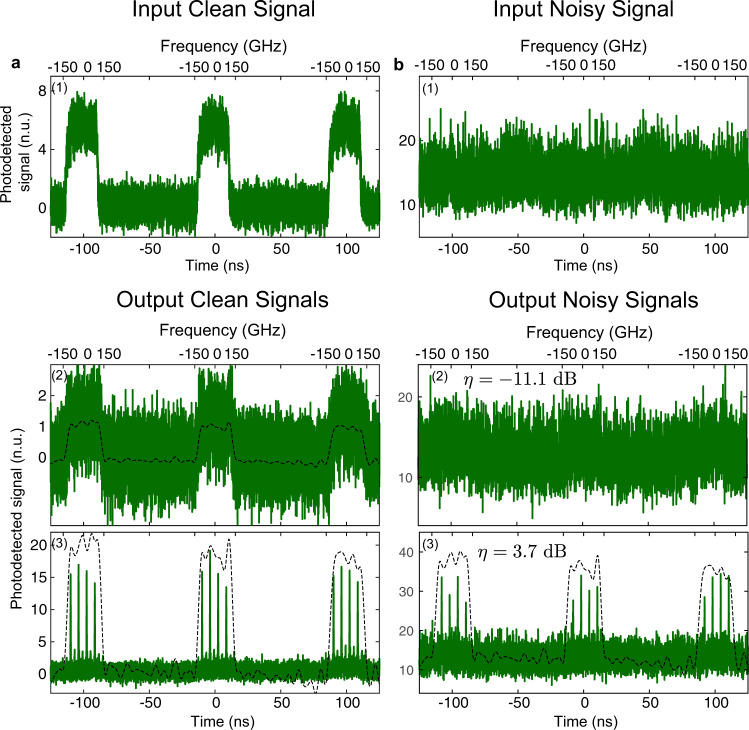
Real-time recovery of spectral waveforms undergoing an S-TAI process. By using real-time optical Fourier transformation (RT-OFT) via dispersive propagation, the spectra of interest were directly mapped into the time domain, such that they could be detected on the fly using a photodiode connected to a real-time oscilloscope (see “Methods”). The measurements are shown in green, and a digitally filtered and scaled version of the SUT spectrum is shown in each case (dashed black) to facilitate a direct comparison with the recovered waveform. All waveforms are normalized to the peak value of the output signal with the phase modulation turned off. **a** (1) A waveform with a relatively high visibility is first generated, and (2) the waveform is attenuated by the insertion loss of the S-TAI processing system, as observed by measuring the waveform at the output of the S-TAI system with the phase modulation off. (3) Activating the phase modulation leads to the formation of spectral peaks, recovering an accurate representation of the initial signal spectrum directly along the time domain (i.e., in real time). **b** Similarly, when the waveform has a low visibility (buried under noise), RT-OFT of the signal at the output of the S-TAI is also able to recover the information of the initial waveform, in a real-time and single-shot fashion.

Using an S-TAI designed with *q* = 20, *ν*_s_ = 2.98 GHz and *ν*_*q*_ = 59.52 GHz, followed by dispersion-induced RT-OFT, isolated single broadband waveforms, centered at an optical frequency of 193.42 THz with a FWHM bandwidth of 250 GHz, were processed and recovered from noise on the fly as they reached the S-TAI system. As observed in Fig. 3a, in the absence of a coherent waveform (e.g., between pulses), the real-time S-TAI scheme simply yields the unaffected noise background. This is a significant advantage as it relieves the need for synchronization, which may be otherwise an important obstacle for the detection of randomly occurring events. To demonstrate the asynchronous capabilities of the S-TAI, the results presented in Fig. 3 were obtained by operating the AWG on its own internal clock, in a completely independent time base from the rest of the signal generation and measurement set-up. This way, the waveform could be measured as it arrives into the detection system using the RT-OFT method employed here. On the other hand, it should be noted that an optical spectrum analyzer (OSA) would fail to record the S-TAI output properly (i.e., when the AWG is not synchronized with the incoming signal), since in this case, the consecutive processed waveforms would not be entirely identical (see Supplementary Fig. 8). The measured amplification of the experimental real-time S-TAI was lower than expected, namely, of 15.79, with measured *ν*_*s*_ = 2.57 GHz and *ν*_*q*_ = 59.58 GHz. We attribute this variation to the limited temporal resolution from the photodetector (PD), as well as the limited available dispersion used for the RT-OFT.

By increasing the amount of noise injected into the system, we could reach a point where the waveform was buried under noise such that no information about it could be retrieved (See Fig. 3b). By comparing the signal without noise (Fig. 3a2) against the noise without the signal present (see Supplementary Fig. 8), the visibility was found to be −11.12 dB. By activating the temporal phase modulation, the real-time S-TAI focused the signal into peaks along the regions where the signal was present, increasing the visibility to 3.7 dB and allowing for real-time detection and recovery of the incoming subnoise waveforms in a single-shot manner.

A key feature of the S-TAI process is that it preserves the spectral phase information of the waveform of interest. This allows to reconstruct the full temporal representation of the waveform. Here we show recovery of waveforms with ~2 ps resolution. Note, however, that there is no fundamental limit to the achievable temporal resolution of the S-TAI, which is only limited by the operation bandwidth of the employed components. On the other hand, as mentioned above, the maximum temporal duration of a signal to be recovered faithfully is given by the inverse of the spectral peak separation, usually limited by the amount of available dispersion. Supplementary Fig. 9 shows a plot of the achievable amplification factor as a function of the sampling rate of the AWG used for temporal modulation and the amount of dispersion. The ratio of the temporal duration of a signal to its fastest variation is given by the time-bandwidth product (TBP), which provides a measure of signal complexity. Waveforms with a TBP of up to 314 were successfully recovered with our S-TAI implementation by processing a waveform with a bandwidth of 5.427 THz, corresponding to temporal variations as fast as ~180 fs (see Supplementary Fig. 10).

**Fig. 4:**
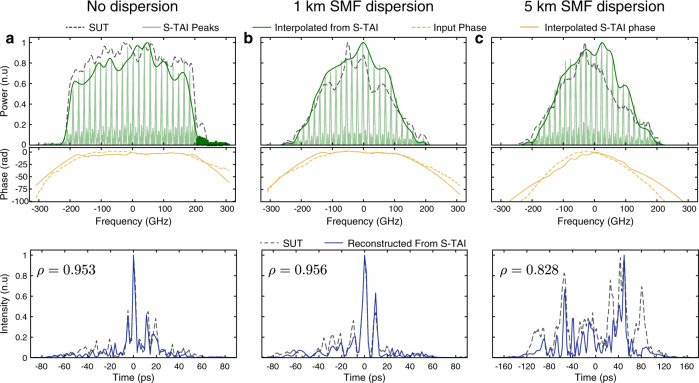
Temporal waveform recovery from the S-TAI output. The temporal waveform can be recovered by interpolating the amplitude and phase from the peaks of the output waveform. The spectral phase profile of each relevant waveform was retrieved using Fourier-transform spectral interferometry (FTSI)^32^. The signals under test (SUTs) are picosecond-resolution waveforms that undergo **a** no dispersion, **b** dispersion after propagation through a 1-km-long section of standard optical fiber and **c** through a 5-km-long section of fiber. (Top) The recovered spectral waveform with (middle) the recovered spectral phase and (bottom) the reconstructed temporal intensity profiles. As measured by the cross-correlation coefficient $\rho$, the recovered S-TAI temporal waveforms show excellent agreement with the measured input temporal waveforms. The S-TAI was designed for *q* = 5, *ν*_*s*_ = 3.5 GHz and *ν*_*q*_ = 17.4 GHz. Each of the shown temporal and spectral intensity waveforms is normalized to unity for clarity.

Employing a combination of the well-known Fourier transform spectral interferometry (FTSI) method and RT-OFT^32^ (see Supplementary Fig. 3), the spectral phase was retrieved to construct the complex-field waveforms for both the unprocessed waveform of interest and the S-TAI (see Fig. 4). By interpolating the amplitude and phase values at the spectral peaks of the S-TAI output, a continuous complex spectral profile was reconstructed and the corresponding temporal waveform was determined by numerical inverse Fourier transform. This allowed for reconstruction of the temporal representation of the waveforms under test with high fidelity, as measured by the Pearson cross-correlation coefficient $\rho$^33^ (see “Methods”). The experiment was carried out for three different input waveforms, distorted by increasing amounts of dispersion, so as to affect both the amplitude and phase profiles of the temporal waveform. When reaching a dispersion corresponding to propagation through a 5-km-long section of standard single-mode fiber (SMF), the temporal waveform was too long to be properly recovered, confirming the expected degradation of the processed signal when the frequency peak separation of the S-TAI is higher than what would be required to adequately represent the features of the waveform spectrum (see “Methods”).

## Discussion

We have developed a versatile method for the detection and full reconstruction of subnoise broadband waveforms that is well adapted to general wave systems. The required components are widely available and the concept is very simple, such that it could potentially be implemented in various practical contexts. By acting on the coherent signal independently of the random incoherent noise, the S-TAI enables the denoising of ultrafast waveforms in a single-shot and real-time fashion, giving access to signals that would be undetectable otherwise. This concept could pave the path for new methods to detect and process signals directly in the spectral domain, allowing for the recovery of previously unattainable information and thus potentially enabling novel and important advances in a wide range of fields, from biology and remote sensing to radio-astronomy.

## Methods

### Theory

The principle behind a TAI is fundamentally based on the perfect autocorrelation property of certain quadratic phase sequences^24^. Thus, the problem can be approached using a discrete formalism to model finite quadratic phase sequences in two Fourier-related domains. These sequences correspond to multi-level phase manipulations in each of the involved signal representation domains, e.g., the frequency and time domains for the S-TAI implementation introduced herein. Although the fundamental principle relies on discrete phase sequences, as derived below, one phase manipulation can be made continuous rather than discrete for experimental convenience, while still implementing the desired TAI effect (the phase manipulation in the other domain should remain discrete). This is employed in the experiments reported here, where a continuous spectral phase filter by GVD (along the frequency domain) is implemented through dispersive propagation, instead of using a multi-level phase filtering scheme.

As illustrated in Supplementary Fig. 1, an S-TAI involves discrete spectral phase filtering of the incoming waveform, followed by discrete temporal phase modulation of the filtered signal. In particular, the transfer function of the filter to be implemented in the first step is composed of a periodic set of spectral phases that change discretely in groups of *q* frequency bins, each of width *ν*_*s*_ such that the *k*th bin, labeled from *k* = 0, …, *q*−1, exhibits the following phase^24^:

4 $$\begin{array}{c}{\phi }_{k{\rm{;}}p,q}={\sigma }_{\phi }\pi \frac{p(1+q{e}_{q})}{q}{k}^{2}.\end{array}$$

Here, *p* and *q* are two mutually prime natural numbers, *σ*_*ϕ*_ = ±1 and *e*_*q*_ describes the parity of the integer *q*, such that *e*_*q*_ = 1 for odd $q$ and *e*_*q*_ = 0 for even *q*. This sequence forms a spectral phase profile in which the discrete phase shifts from Eq. (4) repeat along the frequency axis with a period *ν*_*q*_ = *qν*_s_, where *q* is referred to as the amplification factor. Thus, the bandwidth of the waveform should be at least as broad as the frequency extension of a single complete phase sequence, *ν*_*q*_. As mentioned above, the discrete spectral phase filtering defined by Eq. (4) can in practice be implemented continuously using a second-order dispersive medium (or GVD). In this case, the total second-order dispersion $\ddot{\phi }$ must satisfy

5 $$\begin{array}{c}\ddot{\phi }={\sigma }_{\phi }\frac{p}{q}\frac{1}{2\pi {\nu }_{s}^{2}}.\end{array}$$

It can be easily shown that the quadratic spectral phase variation associated with the amount of dispersion in Eq. (5), applied via the dispersive operator ${{\exp }}\left(\ddot{\phi }{\omega }^{2}/2\right)$, corresponds to the discrete phase shifts defined by Eq. (4) at frequency locations spaced by *ν*_*s*_. Note that the parity term may be dropped since the sequence is no longer truncated to *q* terms. In our designs, *σ*_*ϕ*_ was set to +1, and the integer factor *p* was set to 1 in order to minimize the required amount of dispersion. In general, however, this factor may be set to a different value for an extra degree of flexibility.

As portrayed in Supplementary Fig. 1, the periodic character of the discrete spectral phase filtering process in Eq. (4) will lead to a replication of the original temporal waveform in the time domain, with a time period given by the inverse of the period of the spectral phase function, *t*_*s*_ = 1/*ν*_*q*_. Additionally, the replicated waveforms will exhibit a specific phase variation, such that the *n*th copy will acquire a phase of the form

6 $$\begin{array}{c}{\varphi }_{n{\rm{;}}s,m}=-{\sigma }_{\phi }\pi \frac{s}{q}{n}^{2},\end{array}$$

which is periodic every *q* pulses, and where *s* is also mutually prime with *q*. It has been recently shown that the factor *s* has a deterministic relationship with both *p* and *q* deeply rooted in number theory^24,25^, as described by the relationship

7 $$\begin{array}{c}{sp}=1+q{e}_{q}\left({\rm{mod}}\,2q\right).\end{array}$$

Compensation of the induced temporal phase variation described by Eq. (6) through a suitable temporal phase modulation process (e.g., electro-optic phase modulation, see Supplementary Figs. 2 and 3a) will lead to the coherent addition of the original waveform spectrum into bins of width *ν*_*s*_, separated by *ν*_*q*_.

### Metrics

The values quoted for the FWHM of the output peaks were obtained by fitting a Gaussian by a least-squares algorithm, while the peak separations were taken as the mean value of the distances between adjacent fitted Gaussians.

Concerning the measured amplification values, the quoted values were determined by taking the mean of the ratios of the power of each individual S-TAI peak over the power of the output signal with the phase modulation turned off, measured at the same frequency location. All powers are taken as the average value at the frequency locations of each output peak, relative to the noise floor of the input signal. As further described below in the experimental section, the power values were measured using an OSA with a resolution bandwidth of 140 MHz for all cases reported in the main text.

The visibility, defined in Eq. (3) of the main text, is measured in a similar fashion as the amplification. Thus, the visibility can be understood as a similar metric to the optical signal-to-noise ratio as defined by the IEC 61280-2-9 standard, except that a different resolution bandwidth is employed (e.g., 140 MHz for the OSA measurements reported herein, instead of the conventional 0.1 nm defined by this standard).

The degree of similarity between the recovered temporal waveform and the corresponding input signal was calculated using the Pearson cross-correlation coefficient. This is a widely employed metric for quantitative comparison of real-valued signals and quantifies the similarity between two signals^33^. Specifically, it refers to the maximum value of the cross-correlation of two signals, divided by their autocorrelation at zero lag. For two signals *x*(*t*) and *y*(*t*), the Pearson cross-correlation coefficient is defined as

8 $$\begin{array}{c}\rho =\frac{{\int}_{-{{\infty}}}^{{{\infty }}}x\left(\tau \right)y\left(\tau \right){\rm{d}}\tau }{\sqrt{{\int }_{-{{\infty }}}^{{{\infty }}}{\left|x\left(\tau \right)\right|}^{2}{\rm{d}}\tau {\int }_{-{{\infty }}}^{{{\infty }}}{\left|y\left(\tau \right)\right|}^{2}{\rm{d}}\tau }},\end{array}$$

assuming that they are properly synchronized to ensure that the cross-correlation integral is maximal. For real valued signals, this coefficient takes values between −1 and 1. For two signals satisfying *x*(*t*) = *y*(*t*), up to an offset or scaling factor, the cross-correlation coefficient is *ρ* = 1, whereas if *x*(*t*) = −*y*(*t*), it is *ρ* = −1. The closer this coefficient is to *ρ* = 0, the more dissimilar the signals are.

### Experimental set-up

The basic set-up to implement a S-TAI is shown in Supplementary Fig. 3a. As a dispersive line, the results presented in Fig. 3 and Supplementary Fig. 8 were obtained using a dispersion compensating fiber providing a total second-order dispersion $\left|\ddot{\phi }\right|$ ≈ 936 ps^2^/rad. All other results were obtained using a LCFBG with $\left|\ddot{\phi }\right|$ ≈ 2651 ps^2^/rad, extending over a total frequency bandwidth of 46.30 nm, centered at 1549.2 nm (Proximion). Note that other means may be employed to provide the desired amount of dispersion, such as fiber optic cables or Bragg mirrors. Here, LCFBGs were mainly employed for their high dispersion values, low loss, and compactness. This was followed by a 40-GHz EOPM with a half-wave voltage of 3.1 V at 1 GHz (EOspace). Concerning the phase modulation radio frequency signal generation, the results from the main text presented in Fig. 2 were obtained using an electronic AWG with a sampling rate of 92 Gs/s (Keysight M8196A), the results from Fig. 3 were obtained using an AWG with a sampling rate of 120 Gs/s (Keysight M8194A), and the experiment reported in Fig. 4 was performed using an AWG with a sampling rate of 50 Gs/s (Tektronix AWG70001A).

In all the reported measurements, the optical signal was generated from a 250-MHz mode-locked laser (Menlo systems FC1500-250-WG), which was decimated to 10 MHz using an electro-optic intensity modulator to avoid any interference between consecutive pulses. The signal spectrum was then customized using a programmable WaveShaper (Finisar 4000s) to deliver the different tested optical waveforms with the desired central frequencies, bandwidth, and shapes (see Supplementary Figs. 4–6).

Supplementary Fig. 3b shows the set-up used to obtain the results depicted in Figs. 1 and 2 from the main text. Here, the input signal was combined with the output of a high power EDFA as an ASE noise source, using a 10:90 coupler to maximize the injected noise. The relevant spectra were then measured with a high-resolution OSA (Apex AP2043B) with a resolution bandwidth of 140 MHz. In each of the reported measurements, the spectrum of the input waveform was measured at the output of the S-TAI system, with the temporal modulator off, i.e., after undergoing dispersion only, to account for the practical passive losses of the S-TAI device. Notice that the spectra shown in Fig. 2c–e were renormalized to the shape of the background noise for clarity (see Supplementary Fig. 7 for original data).

In order to measure the single-shot, real-time spectra of the signals of interest, the OSA was replaced by a RT-OFT system^30,31^ (see Supplementary Fig. 3c). By propagating a waveform through a total amount of second-order dispersion ${|\ddot{\phi }}_{{\rm{FT}}}|$, it is possible to perform a Fourier transform directly in the temporal domain, analogous to the spatial phenomenon of Fraunhofer diffraction. Specifically, the spectrum of the input waveform can be mapped along the time domain, i.e., in a real-time fashion, according to the following frequency-to-time mapping law:

9 $$\begin{array}{c}\omega =\frac{{t}_{{\rm{r}}}}{{{\rm{|}}\ddot{\phi }}_{{\rm{FT}}}{\rm{|}}},\end{array}$$

where *ω* is the relative angular frequency variable of the input waveform (around the signal’s central frequency) and *t*_r_ is the relative time variable, with reference to the center of the propagating waveform^31^. This was implemented using a LCFBG with total second-order dispersion ${|\ddot{\phi }}_{{\rm{FT}}}|$ = ${\mathrm{15,581}}$ ps^2^/rad extending over a total frequency bandwidth of 5.32 nm centered at 1547.75 nm (Proximion), corresponding to a mapping rate of 9.72 GHz/ns. This amount of dispersion allows for accurate RT-OFT of waveforms extending over a duration up to *t*_0_ ~ 220 ps, as determined by the following condition^30^:

10 $$\begin{array}{c}{{\rm{|}}\ddot{\phi }}_{{\rm{FT}}}{\rm{|}} \, > \, \frac{{t}_{0}^{2}}{\pi }.\end{array}$$

The time-mapped spectrum was captured with a DC-coupled 9.5 GHz PD (Thorlabs PDA8GS) connected to a 28 GHz RTO (Agilent DSO-X 92804A).

The full-complex field measurement shown in Fig. 4 of the main text was done using balanced FTSI combined with RT-OFT^32^. The idea relies on interfering the signal under test (SUT) with a delayed known reference pulse, creating interference fringes in the spectral domain, from which one can extract the spectral phase profile of the SUT. Using dispersion-induced RT-OFT, the spectral interference profile is mapped into the time domain, such that it can be captured directly with a RTO. The set-up used for this purpose is presented in Supplementary Fig. 3d. A transform-limited optical pulse with a bandwidth of 440 GHz, centered at an optical frequency of 193.342 THz (wavelength of 1550.58 nm) was split into two copies using a 10:90 coupler, where most of the light was used for the SUT, and the remaining portion was used as a reference pulse, whose delay could be tuned using an optical tuneable delay line to set the oscillation period of the interference fringes. The tested SUTs were shaped by filtering them to a bandwidth of 400 GHz using a bandpass filter such that the reference pulse had a bandwidth broader than the SUT. As mentioned in the main text, three different cases were tested to verify that the S-TAI effectively preserves the phase information, by dispersing the SUT through 0, 1, and 5 km of conventional SMF. A second LCFBG with a total second-order dispersion of opposite value as that employed in the S-TAI scheme (that is, same magnitude but opposite sign) was used after the S-TAI device to compensate for the residual phase left from the LCFBG employed for the S-TAI. This ensured an overall non-dispersive propagation along the SUT arm, aside from the SMF utilized for shaping the SUT. Note that this is required due to the continuous nature of the dispersive filtering scheme chosen for this implementation of the S-TAI; this spectral phase compensation step would not be required if a discrete phase filtering scheme was employed instead. The two signals (SUT and reference pulse) were then recombined using a 50:50 splitter to create the interference pattern and split once more by an optical interleaver composed of dispersion-shifted fiber to avoid dispersion effects. This produced two copies of the interference pattern that were *π* shifted with respect to each other. Their spectra were then mapped into the time domain using a LCFBG with total second-order dispersion ${|\ddot{\phi }}_{{\rm{FT}}}|$ = $12,930$ ps^2^/rad allowing for a mapping of 12.4 GHz/ns, with an EDFA to compensate for the losses. After detecting the two waveforms using a 50-GHz PD (Finisar XPDV21x0RA) and the same RTO mentioned above, they were numerically subtracted from one another to isolate the interference pattern. The phase information was then numerically extracted from this interference pattern using the well-known FTSI algorithm^32^. In this calculation, the discrete amplitude and phase profiles of the S-TAI waveform under analysis were made continuous by interpolating the values using a spline, and the temporal waveforms were subsequently determined by numerical inverse Fourier Transform. Considering the peak separation *ν*_*q*_ = 17.4 GHz of the employed S-TAI, this corresponds to maximum temporal durations of ~57.5 ps. This is well satisfied for the 0 and 1 km SMF dispersion cases, for which most of the energy of the corresponding temporal waveforms concentrates well within this duration. As such, the temporal waveform that is recovered through the S-TAI in each of these cases exhibits a high fidelity with respect to the expected one, as confirmed by the resulting high correlation coefficient. On the other hand, the temporal waveform corresponding to the 5 km SMF case clearly extends above the maximum temporal duration specification of the implemented S-TAI. This explains the relatively more significant deviations that are observed between the temporal waveform that is recovered through the S-TAI and the expected one, particularly toward the waveform edges, confirming the anticipated limitations of the S-TAI method.

## Supplementary information

Supplementary Information

Peer Review File

## Data Availability

The data that support the plots within this paper and other findings of this study are available from the corresponding author upon reasonable request.

## References

[1] Horton NG (2013). In vivo three-photon microscopy of subcortical structures within an intact mouse brain. Nat. Photonics.

[2] Ouzounov DG (2017). In vivo three-photon imaging of activity of GCaMP6-labeled neurons deep in intact mouse brain. Nat. Methods.

[3] Liebel M, Toninelli C, van Hulst NF (2018). Room-temperature ultrafast nonlinear spectroscopy of a single molecule. Nat. Photonics.

[4] Chan, C. C. K. *Optical Performance Monitoring* (Academic House, 2010).

[5] Behroozpour B, Sandborn PAM, Wu MC, Boser BE (2017). Lidar system architectures and circuits. IEEE Commun. Mag..

[6] Hankins TH, Kern JS, Weatherall JC, Eilek JA (2003). Nanosecond radio bursts from strong plasma turbulence in the Crab pulsar. Nature.

[7] Marcote B (2020). A repeating fast radio burst source localized to a nearby spiral galaxy. Nature.

[8] Lakowicz, J. R. & Masters, B. R. *Principles of Fluorescence Spectroscopy* (Springer, 2008).

[9] Weiner, A. M. *Ultrafast Optics* (Wiley, 2009).

[10] Vaseghi, S. V. *Advanced Digital Signal Processing and Noise Reduction* (John Wiley & Sons Ltd, 2000).

[11] Tong Z, Radic S (2013). Low-noise optical amplification and signal processing in parametric devices. Adv. Opt. Photonics.

[12] Vasilyev M (2015). Matched filtering of ultrashort pulses. Science.

[13] Wang J (2018). In-band noise filtering via spatio-spectral coupling. Laser Photonics Rev..

[14] Ataie V, Esman D, Kuo BPP, Alic N, Radic S (2015). Subnoise detection of a fast random event. Science.

[15] Maki, H. et al. EEG signal enhancement using multi-channel Wiener filter with a spatial correlation prior. In *Proc. 2015 IEEE International Conference on Acoustics, Speech and Signal Processing* 2639–2643 (IEEE, 2015).

[16] Upadhyay N, Karmakar A (2015). Speech enhancement using spectral subtraction-type algorithms: a comparison and simulation study. Proc. Comput. Sci..

[17] Bozchalooi IS, Liang M (2008). A joint resonance frequency estimation and in-band noise reduction method for enhancing the detectability of bearing fault signals. Mech. Syst. Signal Process..

[18] Ergen, B. In *Advances in Wavelet Theory and Their Applications in Engineering, Physics and Technology* (ed. Baleanu, D.). Ch. 21 (InTech, 2012).

[19] Walden RH (1999). Analog-to-digital converter survey and analysis. IEEE J. Sel. Areas Commun..

[20] Oppenheim, A. V., Willsky, A. S. & Nawab, S. H. *Signals & Systems* (Prentice-Hall, Inc., 1996).

[21] Lohmann AW (1988). An array illuminator based on the Talbot-effect. Optik.

[22] Li B, Li M, Lou S, Azaña J (2013). Linear optical pulse compression based on temporal zone plates. Opt. Express.

[23] Fernández-Pousa CR, Maram R, Azaña J (2017). CW-to-pulse conversion using temporal Talbot array illuminators. Opt. Lett..

[24] Fernández-Pousa CR (2017). On the structure of quadratic Gauss sums in the Talbot effect. J. Optical Soc. Am. A.

[25] Romero Cortés L, Guillet de Chatellus H, Azaña J (2016). On the generality of the Talbot condition for inducing self-imaging effects on periodic objects. Opt. Lett..

[26] Kolner BH (1994). Space-time duality and the theory of temporal imaging. IEEE J. Quantum Electron..

[27] Lukens JM, Leaird DE, Weiner AM (2013). A temporal cloak at telecommunication data rate. Nature.

[28] Crockett, B., Romero Cortés, L. & Azaña, J. Noise mitigation of narrowband optical signals through lossless sampling. in *Proc. of the 2019 European Conference on Optical Communication* 1–4 (ECOC, 2019).

[29] Romero Cortés L, Seghilani M, Maram R, Azaña J (2018). Full-field broadband invisibility through reversible wave frequency-spectrum control. Optica.

[30] Torres-Company V, Leaird DE, Weiner AM (2011). Dispersion requirements in coherent frequency-to-time mapping. Opt. Express.

[31] Azaña J (2000). Real-time optical spectrum analysis based on the time–space duality in chirped fiber gratings. IEEE J. Quantum Electron..

[32] Asghari MH, Park Y, Azaña J (2010). Complex-field measurement of ultrafast dynamic optical waveforms based on real-time spectral interferometry. Opt. Express.

[33] Benesty J, Chen J, Huang Y (2008). On the importance of the Pearson correlation coefficient in noise reduction. IEEE Trans. Audio Speech, Lang. Process..

