# Supplementary material for: Full recovery of ultrafast waveforms lost under noise
Source: Nat Commun. 2021 Apr 23;12:2402. doi: 10.1038/s41467-021-22716-w (PMC8065104; doi:10.1038/s41467-021-22716-w)
Supplement: Supplementary file 1 — Supplementary Information [file 41467_2021_22716_MOESM1_ESM.pdf]

Supplementary Material for:

Full Recovery of Ultrafast Waveforms Lost Under Noise

Benjamin Crockett[1], Luis Romero Cortés[1], Saikrishna Reddy Konatham[1], José Azaña[1,*]

[1]Institut National de la Recherche Scientifique – Énergie Matériaux Télécommunications INRS-EMT, 800 de la Gauchetière Ouest, Suite 6900, H5A 1K6, Montréal (Québec), Canada.

Correspondence: *azana@emt.inrs.ca

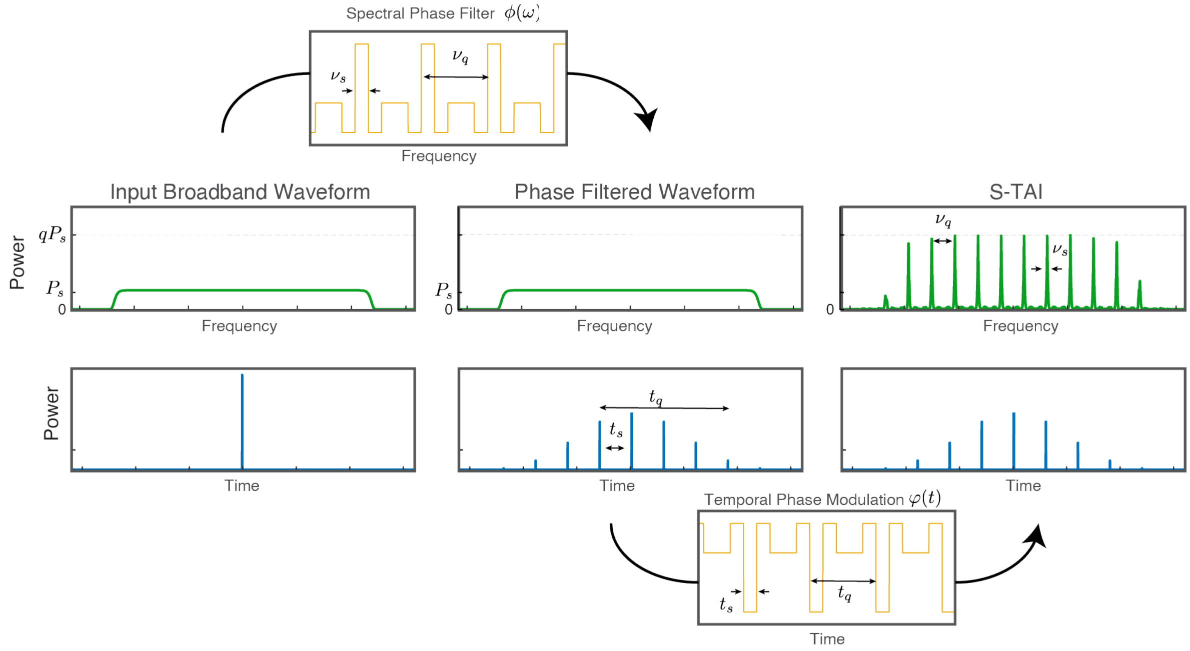

Supplementary Fig. 1 | Operations for a S-TAI. A fully-discrete scheme for the S-TAI is constructed by first filtering an input broadband waveform by a periodic multi-level spectral phase pattern consisting of q levels of length ν_s according to equation (4). The ratio of the signal bandwidth to the total length ν_q of the phase sequence dictates the number of output peaks. In the time domain, this filtered waveform will consist of multiple copies of the input temporal waveform separated by a time t_s, where the nth pulse exhibits a phase according to equation (6), periodic with a repetition period t_q, related to the spectral phase filter pattern through equation (7). Compensation of this temporal phase will lead to the formation of spectral peaks of width ν_s, separated by ν_q, along the frequency domain, outlining the initial spectral waveform amplified by a factor q. For experimental convenience, one of the two phase manipulations can be made continuous rather than discrete, as demonstrated here with the use of group-velocity dispersion (GVD) as a continuous quadratic phase filter in the spectral domain, according to equation (5).

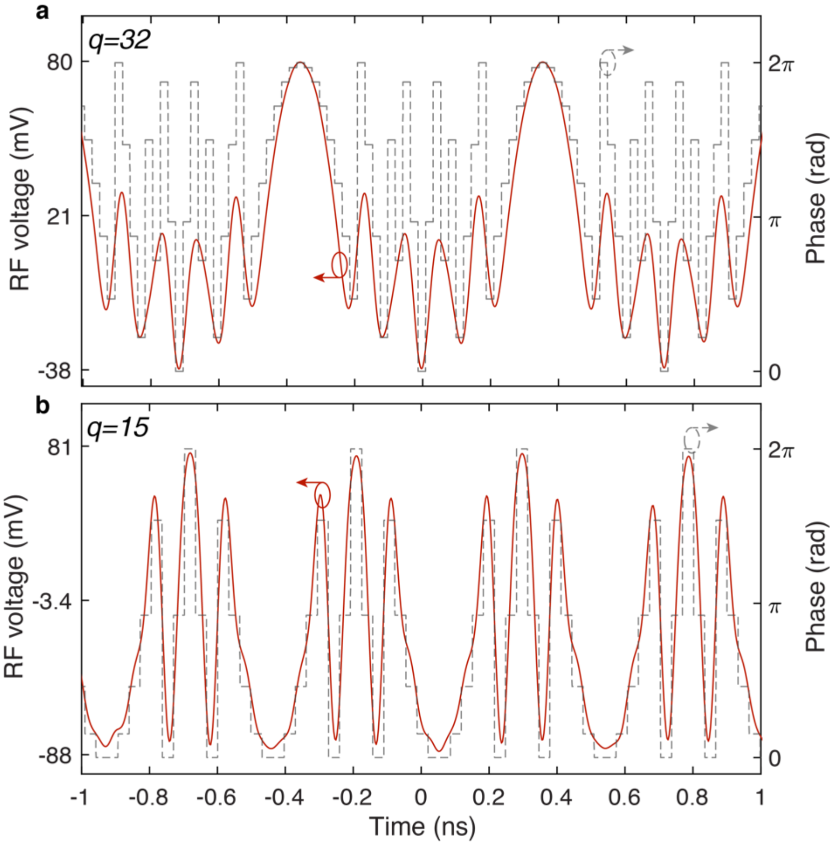

Supplementary Fig. 2 | Temporal phase modulation signal. The ideal phase modulation signal (dashed gray) and measured RF signal (red) used to obtain the results shown in Fig. 2 from the main text, for (**a**) q = 32 and (**b**) q = 15, with corresponding sampling rate ν_s = 44.5 GHz and ν_s = 30.3 GHz, respectively. These were generated using the 92 GS/s AWG, and measured using a 28 GHz oscilloscope. Note that for the q = 32 case, the measurement bandwidth of the scope could not resolve the fast RF signal accurately, which explains the significant discrepancy observed between the theoretical and measured phase profiles.

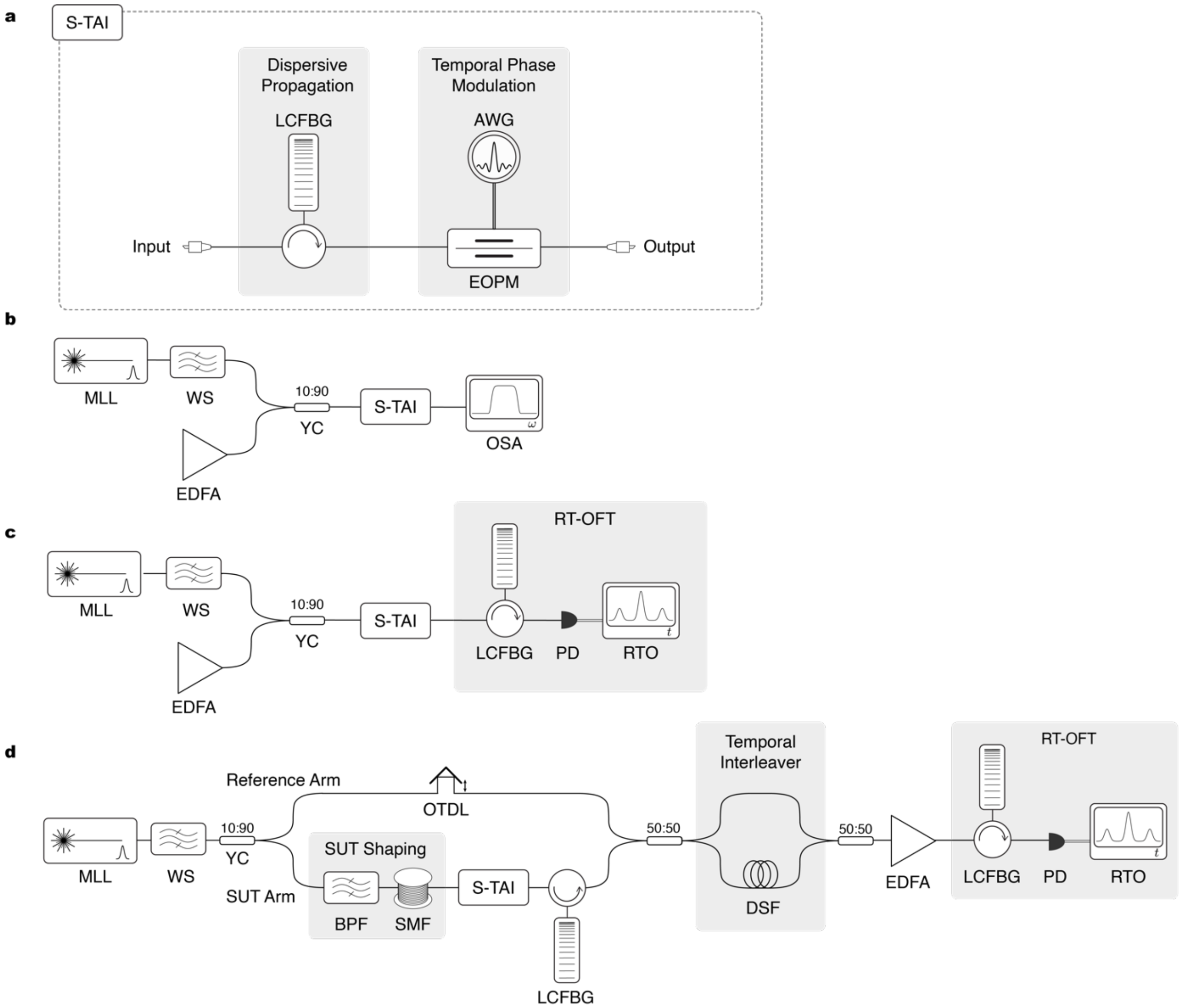

Supplementary Fig. 3 | Experimental setups for the reported demonstrations. LCFBG: Linearly-Chirped Fiber Bragg Grating, AWG: Arbitrary Waveform Generator, EOPM: Electro-Optic Phase Modulator, MLL: Mode-Locked Laser, WS: WaveShaper, EDFA: Erbium-Doped Fiber Amplifier, YC: Y-Coupler, OSA: Optical Spectrum Analyzer, PD: PhotoDiode, RTO: Real-Time Oscilloscope, BPF: BandPass Filter, SMF: Single Mode Fiber, OTDL: Optical Tuneable Delay Line, DSF: Dispersion-Shifted Fiber, Real-Time Optical Fourier Transform (RT-OFT). **a**, The S-TAI implemented here for processing optical waveforms consists of a dispersive medium (LCFBG) followed by a temporal phase modulator (EOPM driven by an electronic AWG). **b**, The S-TAI performance was first measured using an optical spectrum analyzer (OSA) for high spectral resolution and sensitivity. **c**, To show the real-time, single-shot nature of the S-TAI, we implemented a dispersion induced real-time optical Fourier transform system, in which the frequency spectrum at the S-TAI output was mapped along the time domain and captured with a photo-detector attached to a real-time oscilloscope. **d**, The Signal under Test (SUT) temporal waveform was reconstructed from the complex-field (amplitude and phase) spectral waveform, which was measured using a Fourier-Transform Spectral Interferometry (FTSI) system combined with RT-OFT.

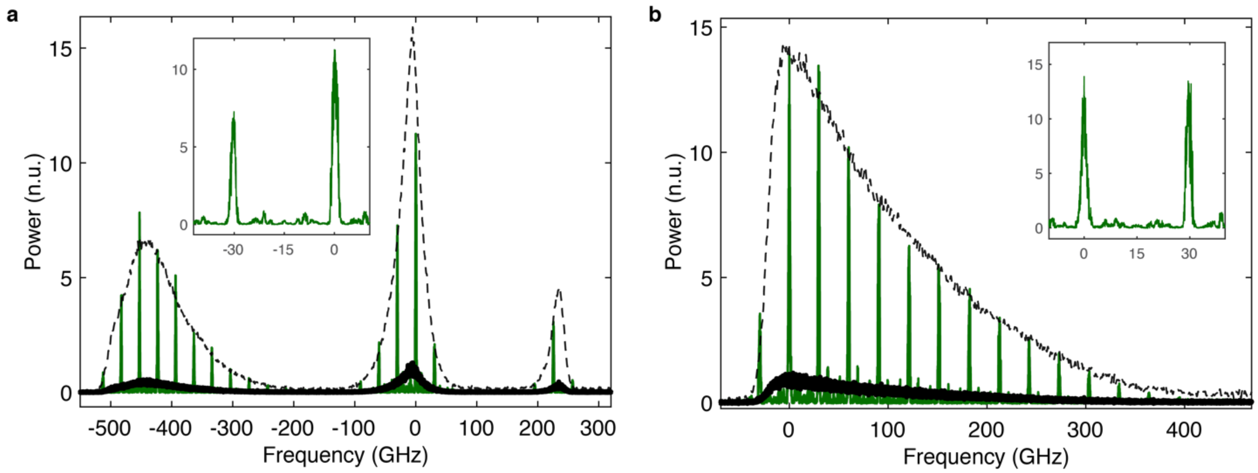

Supplementary Fig. 4 | Spectral Shape Recovery. Signals of different spectral shapes are generated by tailoring the frequency spectrum of a mode-locked laser pulse using a Waveshaper (see Supplementary Fig. 3b). In the two cases reported here, the SUT is measured at the output of the S-TAI processor with the temporal phase modulator turned off, and it is shown using a black trace, the output S-TAI waveform with the phase modulator turned on is shown in green, and a scaled (amplified) copy of the measured SUT is shown using a black dashed line. The inset represents a zoomed-in trace of the output S-TAI waveform, showing the predicted width and separation for the generated spectral peaks. The S-TAI recovers the spectral shape of each signal faithfully, using the same parameters as in Fig. 2b of the main text. All shown optical spectra are represented as a function of the relative frequency with respect to a center optical frequency of (**a**) 193.588 THz (wavelength of 1548.61 nm) and (**b**) 193.224 THz (wavelength of 1551.53 nm).

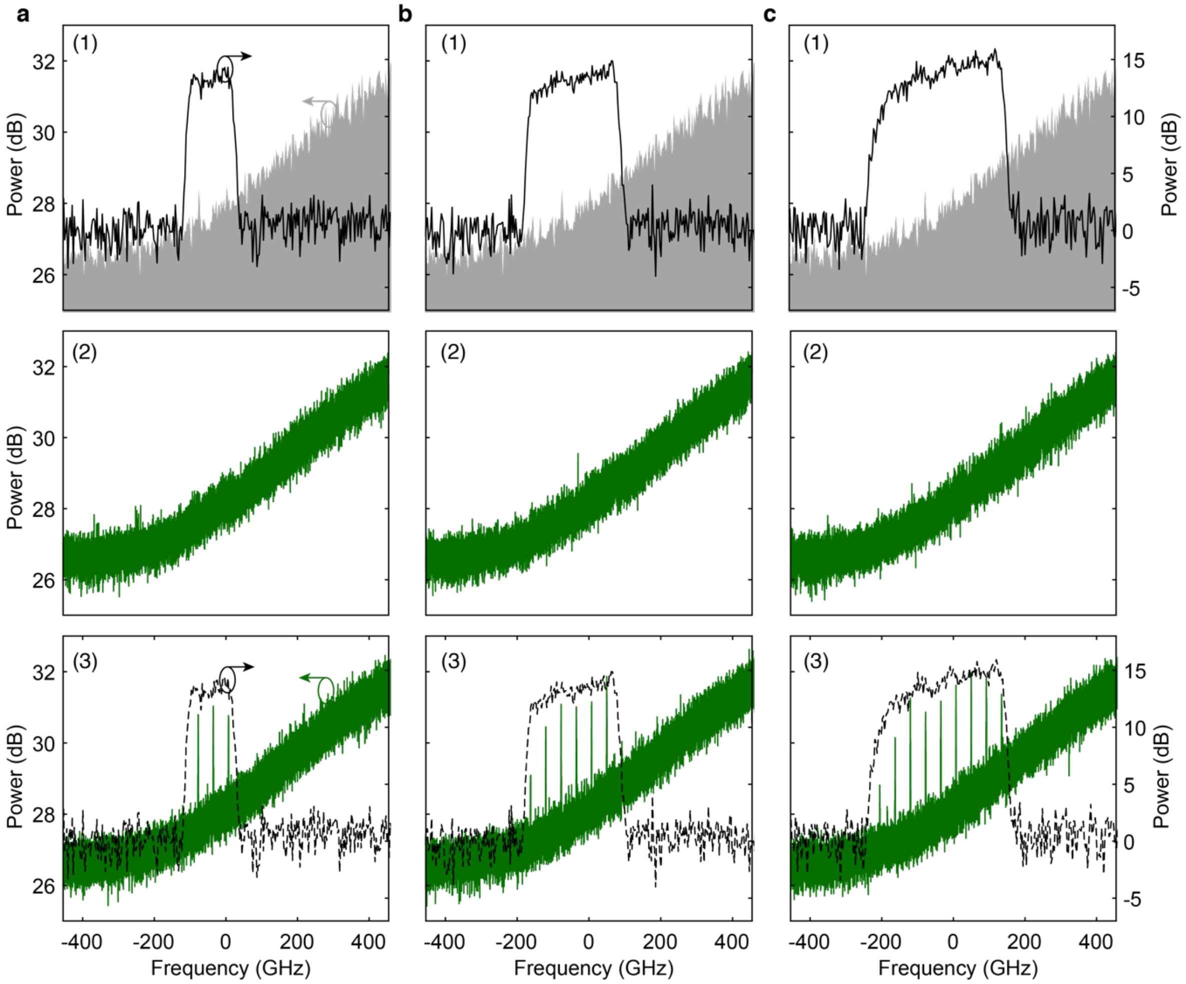

Supplementary Fig. 5 | S-TAI performance for waveforms of different bandwidths. (1) The input waveform of interest (black, right axis) is combined with strong ASE noise (gray, left axis). The shown waveforms are measured at the output of the S-TAI processing system with the phase modulator turned off. (2) The resulting input signal has a significantly degraded visibility, such that the waveform of interest is entirely buried under the noise background. (3) The waveform is recovered using an S-TAI, with the same parameters as for the experiments reported in Fig. 2b of the main text, output signal (with the phase modulator on) shown in green. In these latest figures, the input SUT is shown using a black dashed line, with the corresponding axis on the right, confirming the fidelity of the denoising process. This is done for waveforms centered at an optical frequency of 193.457 THz (corresponding wavelength of 1549.659 nm), for FWHM bandwidths of (**a**) 126.7 GHz, (**b**) 244.3 GHz, and (**c**) 493 GHz.

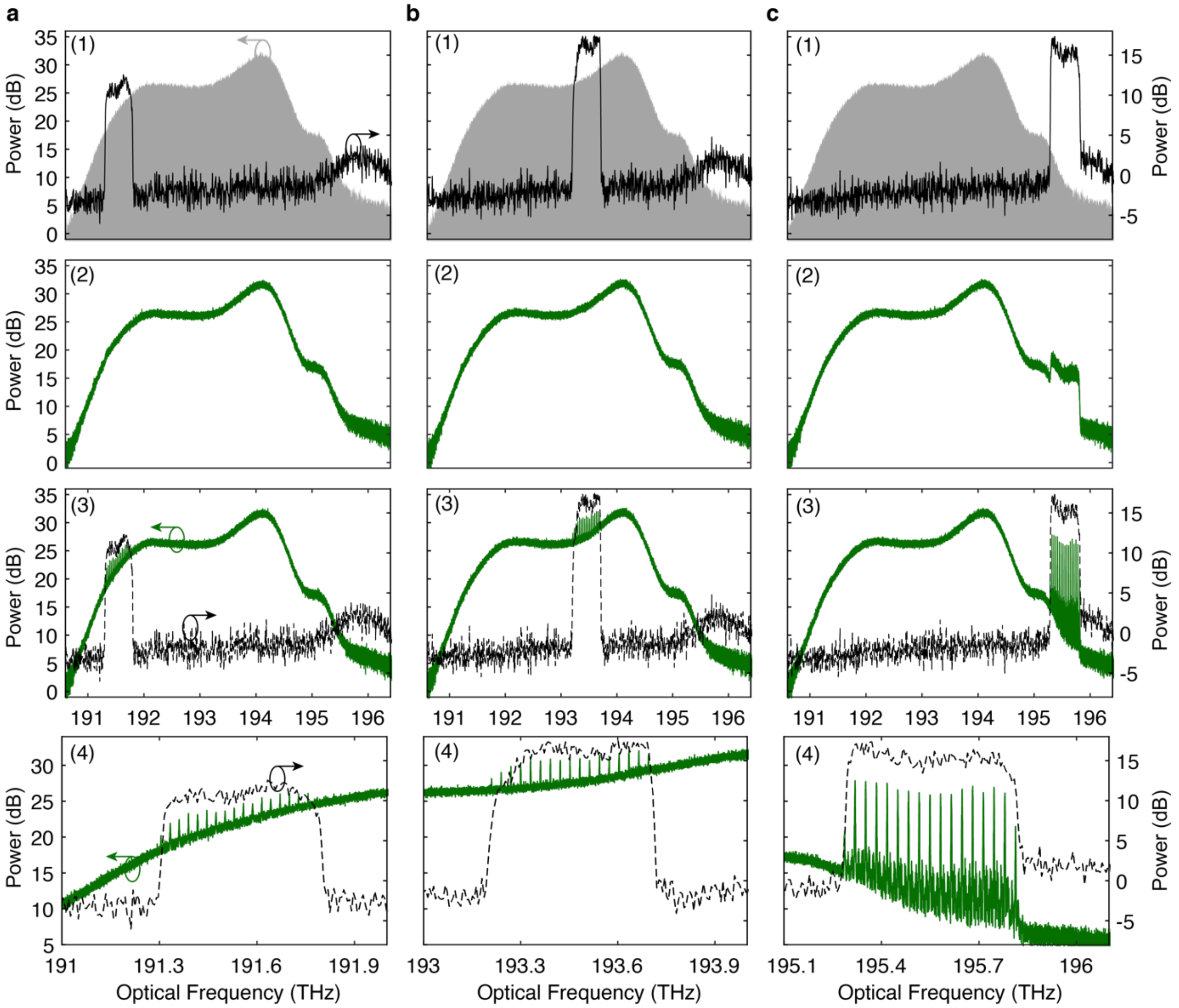

Supplementary Fig. 6 | S-TAI performance for waveforms located at different central frequencies. (1) The input waveform of interest (black, right axis) is combined with strong ASE noise (gray, left axis). The shown waveforms are measured at the output of the S-TAI processing system with the phase modulator turned off. (2) The resulting input signal has a significantly degraded visibility. (3) The waveform of interest is recovered using an S-TAI, with the same parameters as for the experiments reported in Fig. 2b of the main text, output signal (with the phase modulator turned on) shown in green. The input SUT is shown using black dashed line, with the corresponding axis on the right, confirming the fidelity of the denoising process. (4) Close up on the S-TAI output. The reported experiments are performed on waveforms with a FWHM bandwidth of 446.0 GHz, centered at optical frequencies of (**a**) 191.555 THz (1565.046 nm), (**b**) 193.457 THz (1549.659 nm) and (**c**) 195.557 THz (1533.018 nm).

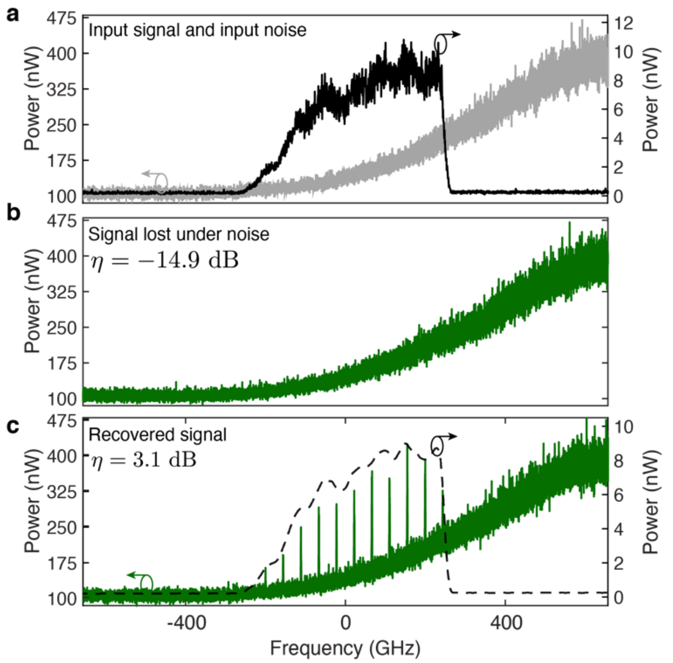

Supplementary Fig. 7 | Noise performance of the S-TAI. Measurements shown in Figs. 2c-e of the main text, with the same design parameters as in the corresponding caption, but without the normalization that compensates for the uneven spectrum of the ASE noise source. **a** A weak optical waveform (black, right axis) is combined with strong stochastic (ASE) noise (gray, left axis). Notice the significant difference in the vertical scales. The shown traces are measured at the output of the S-TAI processing system with the phase modulator turned off. **b**, The waveform is completely buried under noise, becoming undetectable. **c**, Using the same parameters as those in Fig. 2a of the main text, the waveform of interest is successfully recovered from noise (green, left axis), as indicated by η, a measure of the visibility of the waveform against the noise, see equation (3). The input (dashed black trace, right axis) is shown for comparison, confirming that the envelope of the S-TAI peaks outlines an amplified, high-fidelity copy of the input signal.

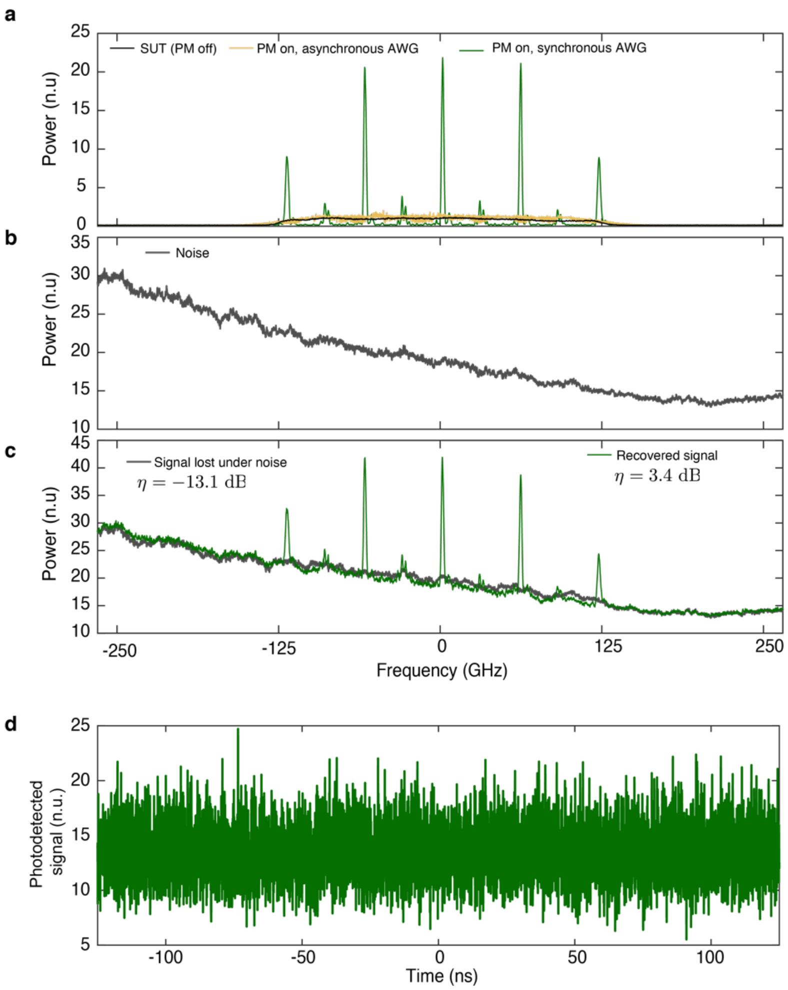

Supplementary Fig. 8 | OSA traces and noise temporal trace for the experimental case analyzed in Fig. 3 of the main article. The results presented in Fig. 3 of the main text were obtained by operating the AWG on its own internal clock, independently of the rest of the signal generation and measurement setup. Since the location of the output spectral peaks depends on the relative time of arrival of the signal and the phase modulation pattern, the resulting amplified waveforms at the S-TAI output were different from one another, such that an OSA could not properly capture the S-TAI peaks. **a** OSA trace of the waveform shown in Fig. 3, measured with a resolution bandwidth of 500 MHz. All values are normalized to the peak of the SUT at the processing system output with the phase modulation turned off. When the AWG is synchronized with the input laser, an amplification factor of 20.3 is measured, with a peak width of 2.57 GHz and peak separation of 60.25 GHz. On the other hand, when the AWG operates asynchronously on its own internal clock (as for the case shown in Fig. 3 of the main text), the peaks are averaged out and no enhancement is observed. **b** Trace of the noise injected in the case analyzed in Fig. 3 of the main text, **c** The input signal is now not visible under the noise background, with a low visibility of η=-13.1 dB. By activating the phase modulator (synchronously), the peaks are now visible with a visibility of η=3.4 dB. **d** Temporal trace of the noise, employed to calculate the visibility for the results shown in Fig. 3 of the main article.

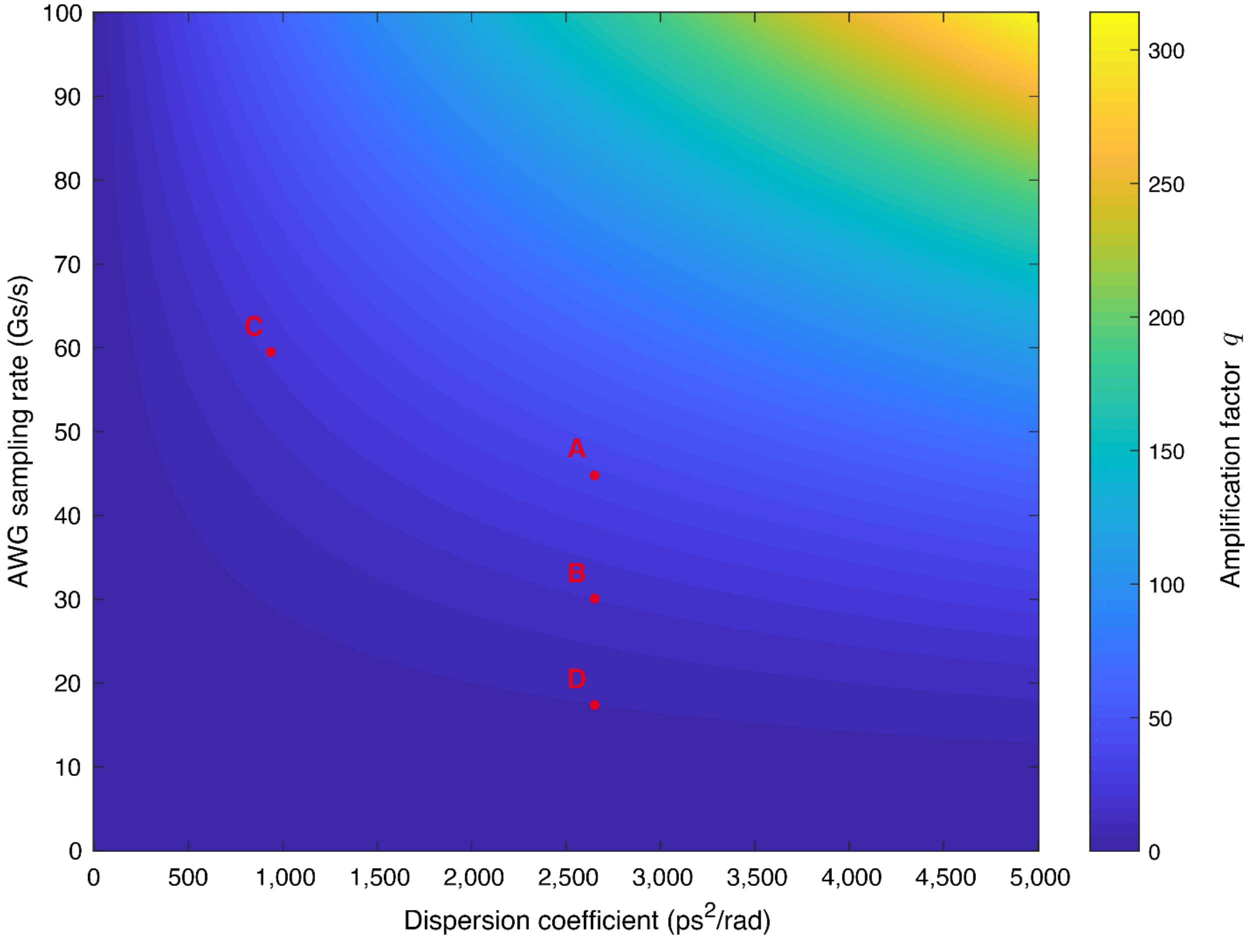

Supplementary Fig. 9 | Amplification factor as a function of the AWG sampling rate and the dispersion coefficient. Available amplification Factor q for a given AWG sampling rate, matching the target spectral peak separation ν_q. Note that this value dictates the longest temporal duration of the SUT that can be recovered through the S-TAI system, determined by $\sim 1/\nu_q$. The red dots indicate the values demonstrated in the reported work: point A is for an amplification of 32, with the S-TAI design specifications corresponding to the results shown in Fig. 2 a and e; point B is for an amplification factor of 15, with the specifications corresponding to the results in Fig. 2 b; point C is for an amplification factor of 20, as per the design specifications corresponding to the results in Fig. 3; and point D for an amplification factor of 5, as per the design specifications corresponding to the results in Fig. 4. As a reference, note that a 10-km long section of a conventional single-mode fiber (SMF-28) introduces a group-velocity dispersion equivalent to about 220 ps^2/rad.

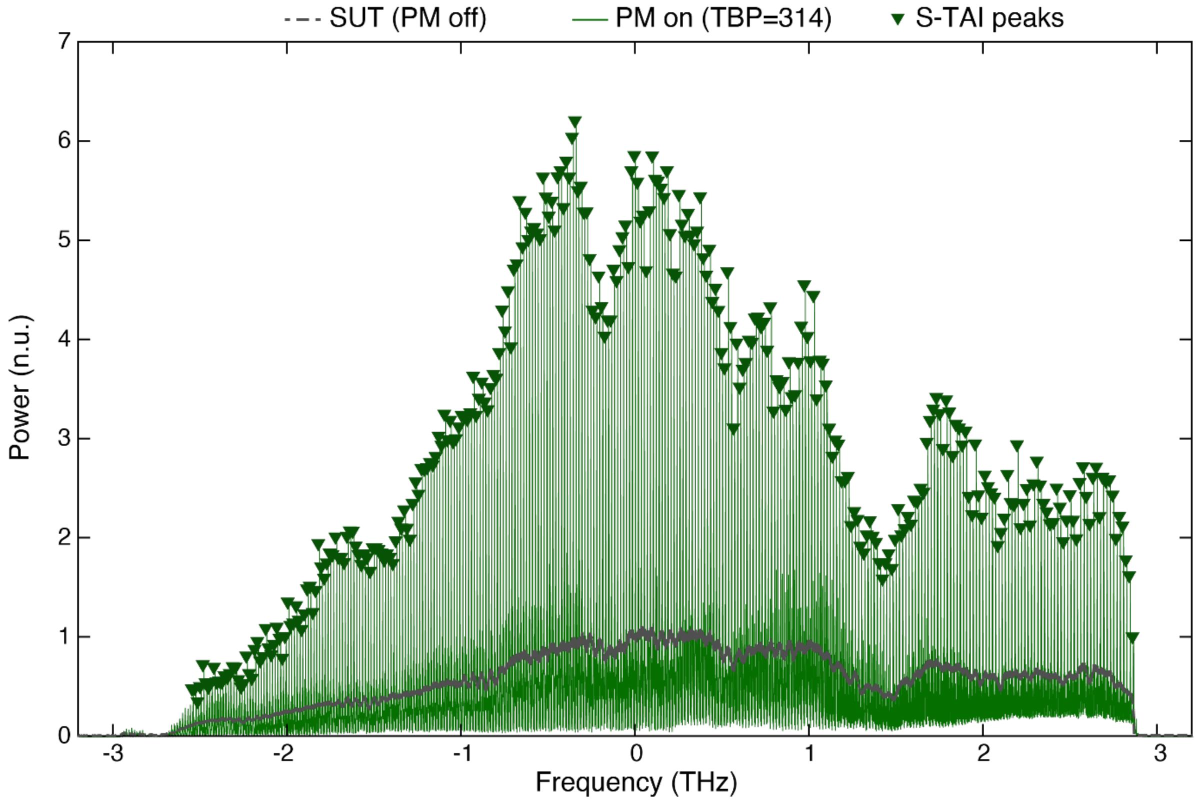

Supplementary Fig. 10 | Time-bandwidth product measurement. The peak separation (ν_q) determines the minimum frequency resolution (inverse of maximum time duration) of the SUT to ensure an accurate full signal recovery. Thus, the spectral sampling process achieved herein enables the recovery of complex signals with a maximum time-bandwidth product (TBP, ratio of bandwidth to frequency resolution) of 314, here shown on a waveform with a 10 dB bandwidth of 5.427 THz, centered at an optical frequency of 193.600 THz (wavelength of 1548.5 nm). The S-TAI was designed for $q = 5$, $\nu_s = 3.5$ GHz, and $\nu_q = 17.4$ GHz, and measured as $q = 4.8$, $\nu_s = 1.9$ GHz, and $\nu_q = 17.2$ GHz.
