# Supplementary material for: Full recovery of ultrafast waveforms lost under noise
Source: Nat Commun. 2021 Apr 23;12:2402. doi: 10.1038/s41467-021-22716-w (PMC8065104; doi:10.1038/s41467-021-22716-w)
Supplement: Supplementary file 2 — Peer Review File [file 41467_2021_22716_MOESM2_ESM.pdf]

Editorial Note: This manuscript has been previously reviewed at another journal that is not operating a transparent peer review scheme. The manuscript was considered suitable for publication without further review at Nature Communications.
